# An Anatomical Variant of Bilateral Persistent Median Artery and Bifid Median Nerve: A Cadaveric Case Report

**DOI:** 10.7759/cureus.66489

**Published:** 2024-08-09

**Authors:** Seetha Aribindi, Eric L Wan, Aditi P Mahajan, A. Lee Dellon, Caitlin R Coker

**Affiliations:** 1 Department of Anatomy, Georgetown University School of Medicine, Washington, DC, USA; 2 Department of Plastic and Reconstructive Surgery, Johns Hopkins University School of Medicine, Baltimore, USA

**Keywords:** bifid median nerve, persistent median artery, carpal tunnel syndrome, anatomical variations, neurovascular variations

## Abstract

An 89-year-old Caucasian male cadaver with prostate cancer demonstrated bilateral persistence of the median artery and bifid median nerve (BMN) during upper limb dissection. The persistent median artery (PMA) originated from the common interosseous artery and coursed alongside the median nerve. Proximal to the carpal tunnel, the median nerve bifurcated into medial and lateral branches.

To our knowledge, this is the first documented case of a bilateral PMA and BMN. While the majority of existing literature focuses on a unilateral PMA or unilateral BMN, bilateral occurrences of either variation are rare. This report presents a novel finding by documenting the simultaneous presence of a bilateral PMA and BMN.

## Introduction

During embryonic development, the median artery plays a critical role in the upper limb arterial system. It typically regresses during week eight of gestation with the formation of the radial and ulnar arteries. However, in some cases, it may persist into adulthood leading to the variation known as the persistent median artery (PMA) [1,2].

Cadaveric studies report notable variability in the prevalence of the PMA ranging from 4% to 27% [1,3]. The literature reports that the PMA is unilateral in 67% of cases and is associated with anomalous median nerves in about 70% of cases, with the most common anomaly being a bifid median nerve (BMN) [4].

In adults, two arterial patterns of the PMA have been documented. In the antebrachial pattern, the PMA is a small, short vessel that ends in the forearm before reaching the wrist, indicating partial involution of the embryonic artery. In the palmar pattern, the artery is larger and longer, traveling with the median nerve through the carpal tunnel to the hand, closely resembling the embryonic arterial pattern [4]. The literature reports that the prevalence of the antebrachial and palmar patterns is 80% and 20%, respectively [4].

A recent study found an 8.3% prevalence rate of the palmar variation of the PMA in patients with carpal tunnel syndrome (CTS) undergoing open carpal tunnel release [5]. A study on population trends in the PMA shows that its prevalence is increasing, suggesting a potential microevolutionary change. It is estimated that the population prevalence of the PMA is 35%, and if current trends continue, the PMA may become a common human structure within the next 80 years [6].

BMN is the division of the median nerve into two distinct nerve bundles in the distal forearm, typically occurring proximal to the carpal tunnel [7]. The prevalence of BMN has been reported to range from 2% to 26% in ultrasound studies [8-12]. To our knowledge, the prevalence of bilateral BMN has not been reported.

The PMA and BMN are anatomical variations with potential clinical significance, particularly in the context of CTS. When considering the combined incidence of the PMA and BMN, the likelihood of encountering both variations in a single individual is significantly lower than for either variation alone. While precise combined incidence rates are not well-documented, the co-occurrence of these anatomical variations can present unique diagnostic and therapeutic challenges. For instance, the presence of a PMA can alter the vascular landscape of the carpal tunnel, potentially increasing the risk of nerve entrapment and impacting the effectiveness of standard CTS treatments. The median nerve is most commonly entrapped in the carpal tunnel; however, other sites of entrapment include the ligament of Struthers, lacertus fibrosus, between the heads of the pronator teres, and the flexor digitorum superficialis. Variations such as a BMN or a PMA can add complexity to these scenarios, necessitating a more nuanced approach to diagnosis and intervention.

To our knowledge, this is the first documented case of a bilateral PMA and BMN. While the majority of existing literature focuses on a unilateral PMA or unilateral BMN, bilateral occurrences of either variation are rare. This report presents a novel finding by documenting the simultaneous presence of a bilateral PMA and BMN. Understanding these variations is crucial for the accurate diagnosis of upper limb pathology and appropriate surgical planning.

This article was previously presented as a meeting abstract and poster at the American Association for Anatomy Meeting on March 22, 2024.

## Case presentation

**Figure 1:**
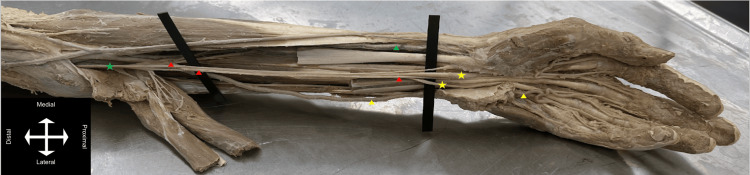
Anterior view of the left forearm displaying the relationship between the PMA and BMN. Yellow star = BMN (medial and lateral branches); red arrows = PMA (origin, proximal, and distal); green star = median nerve (proximal); green arrow = radial artery; yellow arrow = ulnar artery (proximal and distal as it contributes to the palmar arch). PMA = persistent median artery; BMN = bifid median nerve

**Figure 2:**
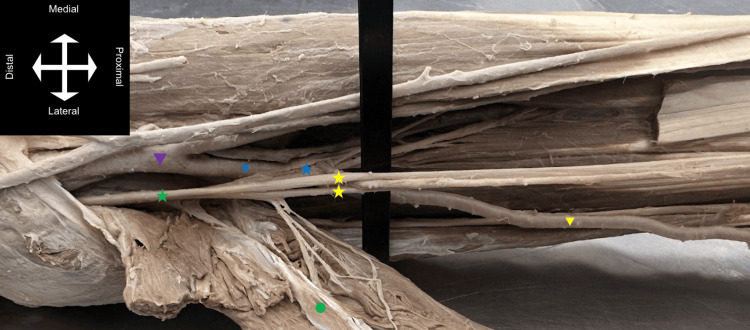
Anterior view of the left forearm displaying the relationship between the PMA and BMN. Purple arrow = brachial artery; blue arrow = common interosseous artery, the origin of the PMA as it courses through the branches of the anterior interosseous nerve (blue star), and pierces the median nerve (green star) forming the BMN (yellow stars); green circle = superficial anterior forearm muscles; yellow arrow = ulnar artery. PMA = persistent median artery; BMN = bifid median nerve

**Figure 3:**
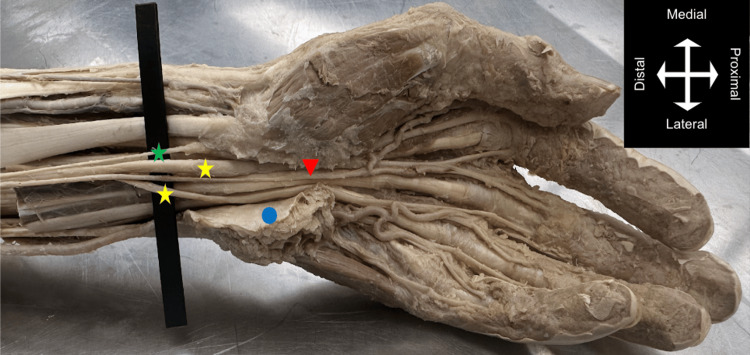
Anterior view of the left distal forearm and hand demonstrating the PMA coursing between the branches of the BMN into the carpal tunnel. Green star = median nerve (palmar branch); yellow stars = BMN (lateral and medial branches); red arrow = PMA contributing to palmar arch; blue circle = flexor retinaculum. PMA = persistent median artery; BMN = bifid median nerve

This case presentation was performed in accordance with the requirements of the Declaration of Helsinki. During the routine cadaveric dissection of an anatomical donor at the Georgetown University School of Medicine, an 89-year-old Caucasian male donor demonstrated a bilateral PMA and BMN. Bilaterally, the PMA originated from the common interosseous artery, traveled distally through the anterior forearm and carpal tunnel, and terminated in the hand (Figure 1). Proximally, near its origin, the PMA punctured branches of the anterior interosseous nerve and then traveled superficially to pierce the median nerve (Figure 2). In the distal two-thirds of the forearm, the PMA traveled anterior to the median nerve, traversing the space between the flexor digitorum superficialis and the flexor digitorum profundus muscles. Proximal to the carpal tunnel, the median nerve bifurcated bilaterally into its medial and lateral branches. The PMA traveled between the branches of the BMN through the carpal tunnel and into the hand (Figure 3). In the hand, the PMA coursed radially, aligned with the lateral branch of the median nerve, which innervated digits 1, 2, and the lateral half of digit 3. The PMA gave rise to an incomplete superficial palmar arch configuration, as there was no anastomosis between the regions supplied by the median and ulnar arteries in the hand (Figure 3). During our dissection, we observed that both the ulnar and radial arteries followed their typical anatomical pathways without any deviations. The ulnar artery originated from the brachial artery at the cubital fossa. It then traveled down the medial side of the forearm, passing deep to the flexor muscles. In the wrist, it entered the hand through Guyon’s canal, terminating in the superficial palmar arch, which supplied blood to the hand. Similarly, the radial artery originated from the brachial artery at the cubital fossa. It ran down the lateral aspect of the forearm, passing beneath the brachioradialis muscle. At the wrist, it traveled through the anatomical snuffbox and then entered the hand and contributed to the deep palmar arch, providing blood supply to the hand (Figure 3).

## Discussion

Bilateral occurrences of both a PMA and a BMN are rare, and, to our knowledge, this is the first documented case. Existing literature primarily focuses on unilateral PMA, unilateral BMN, or unilateral concomitant PMA and BMN. This novel finding, combined with the importance of understanding anatomical variations to improve surgical planning and develop a comprehensive understanding of arm anatomy, makes this case a significant contribution to current literature. In a 2017 study, Chen et al. examined 160 wrists and found that 15 (9.4%) wrists had a BMN, and 12 (7.5%) wrists had a PMA. These anatomical variations could occur together or independently. Notably, the probability of both variations coexisting (6.3%) was higher than the probability of having only a BMN (3.1%) or only a PMA (1.3%) [13].

Understanding the topography of hand anatomy is crucial. As the most mobile part of the body and essential for most tasks, the hand is frequently exposed to injury. Therefore, recognizing the relationships and variations of different vessels in the hand and forearm, including their diameters and anastomoses, is vital for vascular and microvascular surgery in this area [13].

The presence of a PMA, which may not always be evident as many patients are asymptomatic, has been linked to clinical consequences related to CTS [14]. Srivastava et al. described a case of a 38-year-old male patient who presented with CTS-like symptoms and thrombosis of the PMA. Consistent with our findings, the PMA in this patient traveled through the carpal tunnel adjacent to the median nerve, and the thrombosed artery caused compression neuropathy. The administration of intravenous heparin therapy resulted in clinical improvement of symptoms [14]. This presentation mirrors a case reported by Salter et al., involving a 42-year-old female patient with wrist pain and CTS-like symptoms due to a thrombotic PMA that compressed the median nerve, exacerbating CTS [15]. While we did not specifically observe thrombosis of the PMA in our cadaver, surgeons should consider the possibility of a PMA thrombosis and use ultrasound imaging to rule out the condition as needed.

Other studies have found that the presence of a PMA, when it extends into the carpal tunnel, is implicated as a potential cause for CTS [2,5]. In 2019, Haladaj et al. described the prevalence and variability of the PMA configurations in the general population. After dissecting 125 randomly selected, isolated upper limbs, Haladaj et al. reported a 4% prevalence of PMA. They noted that the PMA can branch from the ulnar artery, anterior interosseous artery, or common interosseous artery. This study emphasized that a PMA may contribute to CTS-related pain due to the carpal tunnel’s inability to expand [1].

Any structure occupying space within the carpal tunnel can compress the median nerve, leading to compressive neuropathy. The specific topography of the PMA and the median nerve can alter their orientation inside the carpal tunnel in various ways. Two patterns of the PMA have been identified in the literature. In the first pattern, the PMA originates from the common caudal angle between the ulnar artery and the common interosseous trunk, anastomosing with the ulnar artery in the hand to form the superficial palmar arch. In the second pattern, observed in our cadaver, the PMA originates from the anterior interosseous artery, does not anastomose in the palm, and does not form the superficial palmar arch [2]. Both patterns can occupy space within the carpal tunnel, potentially causing or exacerbating CTS through direct compression of the median nerve, thrombosis, or aneurysm. The variability in presentation highlights the importance of preoperative ultrasounds to avoid iatrogenic injuries. This information is also crucial when the hand is exsanguinated and under tourniquet control, as potential arterial damage or local hemorrhage may not be identified until the tourniquet is released postoperatively [2].

Recent literature has found that the prevalence of PMA is likely higher in patients with CTS. In a 2022 study, Townsend et al. analyzed 327 patients undergoing open carpal tunnel release. They reported a PMA prevalence rate of 8.3% and a BMN prevalence rate of 0.6%. These findings further underscore the importance of including a PMA and a BMN assessment in ultrasound evaluations for CTS [5].

Additionally, the literature has reported that the presence of a BMN may be implicated in patients with CTS. In a 2016 study, Narayan et al. reported a case of a 40-year-old male patient with CTS. They found that a BMN may be more common in patients with CTS, likely due to the larger cross-sectional area occupied within the carpal tunnel compared to those with a normal median nerve anatomy. This additional volume may contribute to increased compression within the carpal tunnel, exacerbating CTS symptoms [7].

## Conclusions

To our knowledge, this is the first report of a bilateral PMA and BMN. While the majority of existing literature focuses on a unilateral PMA or a unilateral BMN, bilateral occurrences of either variation are rare. This report presents a novel finding by documenting the simultaneous presence of a bilateral PMA and BMN, offering new insights into how these anatomical variations might interact and manifest in clinical scenarios.

Current literature suggests that the PMA is an anatomical variation with increasing prevalence. Given its association with CTS symptoms, it is crucial for plastic and orthopedic surgeons to be aware of this variation to minimize potential damage during CTS procedures. Additionally, ultrasound has been identified as an effective tool for detecting the PMA and the BMN, aiding in accurate surgical planning, especially for minimally invasive hand procedures. Further research is needed to determine more precise prevalence rates for the PMA and BMN and to explore their respective patterns in the upper limb.

## Figures and Tables

**Figure 4:**
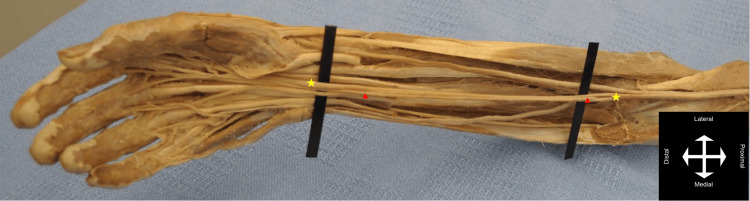
Anterior view of the right forearm displaying the relationship between the PMA and BMN. Yellow star = BMN (proximal and distal); red arrow = PMA (proximal and distal). PMA = persistent median artery; BMN = bifid median nerve

**Figure 5:**
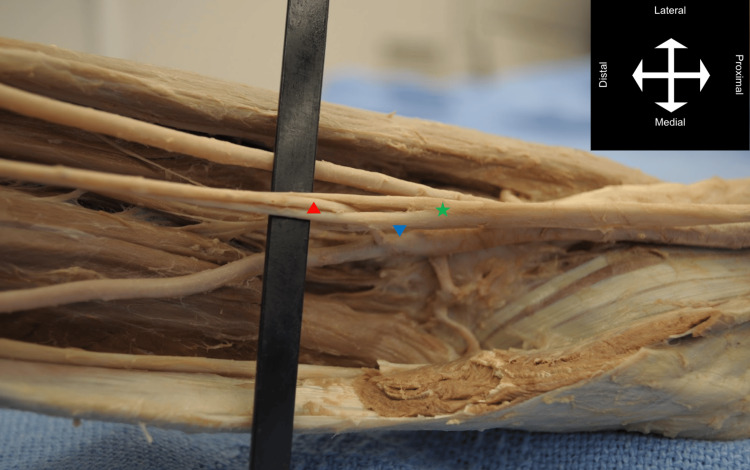
Anterior view of the right forearm displaying the relationship between the PMA and BMN. Red arrow = PMA as it originates from the common interosseous artery (blue arrow).; green star = PMA as it pierces the median nerve proximally. PMA = persistent median artery; BMN = bifid median nerve

**Figure 6:**
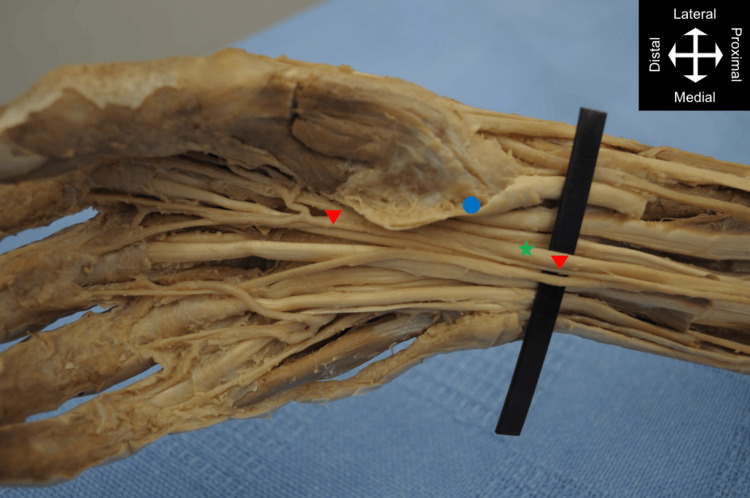
Anterior view of the right distal forearm and hand demonstrating the PMA coursing into the carpal tunnel. Red arrows = PMA proximally as it contributes to the palmar arch; blue circle = flexor retinaculum; green star = median nerve PMA = persistent median artery; BMN = bifid median nerve
